# Numerical Study on the Stomatal Responses to Dry-Hot Wind Episodes and Its Effects on Land-Atmosphere Interactions

**DOI:** 10.1371/journal.pone.0162852

**Published:** 2016-09-20

**Authors:** Shu Wang, Hui Zheng, Shuhua Liu, Yucong Miao, Jing Li

**Affiliations:** 1 Department of Atmospheric and Oceanic Science, Peking University, Beijing, China; 2 Key Laboratory of Regional Climate-Environment Research for Temperate East Asia, Institute of Atmospheric Physics, Chinese Academy of Sciences, Beijing, China; 3 Department of Geological Sciences, John A. and Katherine G. Jackson School of Geosciences, University of Texas in Austin, Austin, Texas, United States of America; University of Western Sydney, AUSTRALIA

## Abstract

The wheat production in midland China is under serious threat by frequent Dry-Hot Wind (DHW) episodes with high temperature, low moisture and specific wind as well as intensive heat transfer and evapotranspiration. The numerical simulations of these episodes are important for monitoring grain yield and estimating agricultural water demand. However, uncertainties still remain despite that enormous experiments and modeling studies have been conducted concerning this issue, due to either inaccurate synoptic situation derived from mesoscale weather models or unrealistic parameterizations of stomatal physiology in land surface models. Hereby, we investigated the synoptic characteristics of DHW with widely-used mesoscale model Weather Research and Forecasting (WRF) and the effects of leaf physiology on surface evapotranspiration by comparing two land surface models: The Noah land surface model, and Peking University Land Model (PKULM) with stomata processes included. Results show that the WRF model could well replicate the synoptic situations of DHW. Two types of DHW were identified: (1) prevailing heated dry wind stream forces the formation of DHW along with intense sensible heating and (2) dry adiabatic processes overflowing mountains. Under both situations, the PKULM can reasonably model the stomatal closure phenomena, which significantly decreases both evapotranspiration and net ecosystem exchange of canopy, while these phenomena cannot be resolved in the Noah simulations. Therefore, our findings suggest that the WRF-PKULM coupled method may be a more reliable tool to investigate and forecast DHW as well as be instructive to crop models.

## Introduction

At present, boosting world population growth has further intensified the food shortage [1]. Climate has fundamental impacts on natural terrestrial ecosystems and cultivated agricultural ecosystems by means of environmental temperature, radiation, moisture, atmospheric constituents, and weather or climate extremes [2, 3]. The nexus between cropland ecosystems and climate needs clarification. Enormous researches based on statistics of abundant data and state-of-the-art numerical modeling analyze or project agricultural systems’ response to climate in various ways, but uncertainty still remains [4, 5]. It should be paid attention to that ecosystems bear increasing vulnerability induced by more frequent agro-meteorological disasters resulted from climate variation and change [6–10].

Water, heat, and solar radiation are the key ambient factors for crop growth. According to enormous evidence of crop cultivation experiments and model simulations, daily thermal condition dominates crop development in the form of accumulated temperature and agricultural disasters [11]. Many cases of ecosystem productivity depletion caused by heat wave along with water scarcity have been reported world widely [12, 13]. The common methodology for heat wave statistics is setting thresholds of mean temperature or combination of maximum and minimum temperature [14, 15]. As for vast rain-fed croplands in north and northwest interior China, water scarcity either in meteorology or hydrology, is the top restriction factor for ecosystem and agriculture [16, 17]. Observed temperature rises have extended plantable growing seasons and seeding area in China [18] which leads to more water deficitin, for instance, vast arid Northwest China.

Wheat is one of the most important food crops in China (wheat cropland occupies 20% of total tillage in area). The major threat to wheat yield is the heat wave along with low humidity and certain wind speed during premature grain-filling season of wheat. Chinese agronomists define a combined metric for this kind of disaster named dry-hot wind (hereafter, DHW) based on empirical observation. On basis of former researches on distinguishing occurrence time of DHW, one slight (severe) DHW episode is defined when the daily maximum temperature exceeds 32°C (35°C), relative humidity at 1400 at Local Standard Time (LST) is lower than 30% (25%), and wind speed at 1400 LST exceeds 2 m/s (3 m/s). High temperature, low humidity and strong wind will directly lead to intensive evapotranspiration, water unbalance in plant, enzyme sabotage, inhibition of grain carbohydrate synthesis, and then yield reduction. Based on agronomic statistics throughout decades, DHW can cause a significant large-area grain reduction by 10~20% for pre-mature wheat at our region of interest [19]. According to records, local DHW statistically begins from May 10, and the disaster magnitude increases as the temperature rise. The occurrence frequency of DHW is increasing recently over China caused by climate warming. Therefore, the study of DHW variance and its terrestrial interaction features over this agricultural area is theoretically and practically necessary.

Moreover, due to the mass production of higher planting density, crop yields are affected by DHW more prominently. Irrigation is the only way to mitigate DHW threat, but we need to verify irrigation timing and amount of water consumption to improve irrigation efficiency [20]. Physiological observation and corresponding simulation of DHW are imperative so far since most domestic studies are statistical. Further understanding on its micrometeorological and physiological mechanism is vital to mitigation this kind of agricultural disaster.

In soil–plant–atmosphere continuum, the interactions between land surfaces and the overlying air are by means of exchanges of heat, moisture, trace gases, and momentum [21]. Land–atmosphere interactions are largely regulated by turbulence in atmospheric surface layer. Land-surface climate is characterized as stable systematic features of diurnal and seasonal cycle [22]. Heat and moisture flux are the crucial parts of terrestrial interactions, which link the energy balance and water utility [23]. Besides, crop yields [24] and vegetation evolution under global climate change [25, 26] are high relevant to heat and water exchange.

To resolve this flux explicitly, the land surface model (LSM for short) is one of the most reliable approaches. LSMs describe the physical processes of radiation, turbulent transfer, soil heat and water transfer, plant physiology at the interface of land and atmosphere. A fundamental function of LSM is the energy partitioning for mesoscale models or general circulation models. LSMs have evolved rapidly in recent years with improved parameterizations and incorporation of new biogeochemical processes, on basis of lots of indispensable land surface physics processes experiments [27–29]. Ample complicated parameterizations have been introduced into the state-of-art third-generation LSMs, along with more detailed interpretation of physical, ecological and chemical processes embedded [30, 31]. Hereby, we focus on the terrestrial water and energy flux budget over a wheat land during DHW processes with extensive heat, kinetic energy and water exchange, from a land-surface physical process view. Thus LSM is a preferred tool. A third-generation LSM developed by Peking University is presented in this paper. The model performance on flux and soil characteristics simulation has been verified in semi-arid area [32].

In this paper, we investigate the characteristics of near-surface micrometeorology, terrestrial interactions and reveal the plants’ physiological responses to DHW over cultivated wheatland on Guanzhong Plain located in midland China, by using Weather Research and Forecasting (WRF) and Peking University Land Surface Model (PKULM). In next section, we introduce the data, geographical information followed by model configuration and PKULM model implementation. Simulation results and analysis are presented then, with discussion and conclusions.

## Data and Model Description

### Meteorological and satellite data

Hourly temperature, relative humidity, dew point temperature, wind speed data derived from weather stations at Wugong County from 2008 to 2014 are processed and analyzed. For lack of relative humidity record, relative humidity is calculated based on Tetens formula [33]. Statistically, there are 5, 8, 1, 6, 6, 7, 4 days when DHW episode occurred and/or sustained respectively from 2008 to 2014, according to the metrics previously mentioned. Among them, two DHW episodes in 2011 and 2013 are both severe and continuous. Obvious high DHW occurrence frequency is a notable threat to food security. Later in this article, we focus on the investigation of 2011 and 2013 DHW episodes. The detailed information of timing, meteorological factors during the two DHW episodes are presented in Table 1.

**Table 1:** DHW occurrences at Wugong in June, 2011 and May, 2013, with factor values and disaster grade.

Time	Daily maximum temperature (°C)	Relative humidity at 1400 LST (%)	Wind speed at 1400 LST (m s^-1^)	Disaster grade
2011/06/06	36.2	28.2	3	Severe
2011/06/07	39.8	26.9	1	Null
2011/06/08	37.5	25.5	3	Severe
2013/05/20	34.1	30.0	2	Slight
2013/05/21	38.1	29.7	2	Slight
2013/05/22	34.9	24.6	2	Slight

Using remote sensing data to obtain parameters for LSM is an effective approach to determine some representative values for grid scale. The monthly-averaged LAI (Leaf Area Index) prescribed in Noah LSM in this case is 1, which is supposed to be underestimated. In this simulation, a series of real-time LAI data derived from FY-3 satellite is utilized to characterize surface vegetation. Hereby we employ 3.78 (an averaged LAI in-situ extracted from FY-3 product, see S1 Fig) as the LAI value in the plot-scale simulation during 2013 May DHW episode. The satellite data processing approach and its reliability have been verified by Zhu et al [34]. We average LAI value over pixels of cultivated and managed farmland category according to the land use category list from Global Land Cover Characterization [35].

As mentioned before, the two LSM models differ in many respects. The difference in LAI calculation thus need to be clarified hereby. Typical application of Noah in mesoscale models adopts grid sizes of kilometers square or so. The calculation of the LAI is based on fixed monthly vegetation coverage. Formula (19) is utilized to estimate LAI value on each model grid [36]:

$$LAI=\frac{\sigma_{f}-\sigma_{f,min}}{\sigma_{f,max}-\sigma_{f,min}}\left(LAI_{max}-LAI_{min}\right)+LAI_{min}$$ (1)

Where *σ*_*f*,*max*_ and *σ*_*f*,*min*_ are the maximum and minimum vegetation coverage valued at 0.96 and 0.01 respectively. *LAI*_*max*_ and *LAI*_*min*_ are the maximum and minimum LAI, valued 6 and 1 respectively [37]. While in this PKULM modeling, LAI is set to 3.78 based on FY-3 satellite 8-day average LAI product. Since the vegetation coverage changes rapidly in certain months, low temporal resolution of LAI dataset can not guarantee the LAI value utilized in Noah reliable to reflect the real physiological condition of canopy. Consequently, Adoption of LAI dataset with higher spatial and temporal resolution will produce more reliable parameters for model configuration, such as ground albedo and roughness.

### Geographical information about the study area

**Fig 1:**
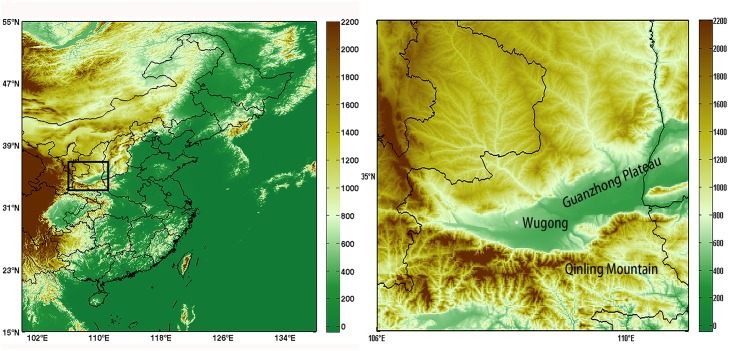
Location and terrain scope of Guanzhong Plain.

Our study area is the Guanzhong Plain located in midland China (Fig 1). East Asia Monsoon dominates the climatological background, while the regional climate is attached to geography. As shown in Fig 1, geographically this plain is surrounded by mountains. Noted that the Qinling Mountain located in the south is 2000–2500 m elevation upgrade from the plain (about 300m above sea level). However, Qinling Mountain hinders seasonal moist and warm air from going northward, which possibly leads to the formation of Foehn and precipitation inhibition in the downwind area over the mountain. From another respect, there is a “gap tube” in the western entrance of the plateau. Once hot, dry northwest wind blows into above the plateau, the near-surface wind speed might increase correspondingly. Specifically how topography affects DHW disaster will be revealed via simulation in the following part.

### WRF configuration

**Fig 2:**
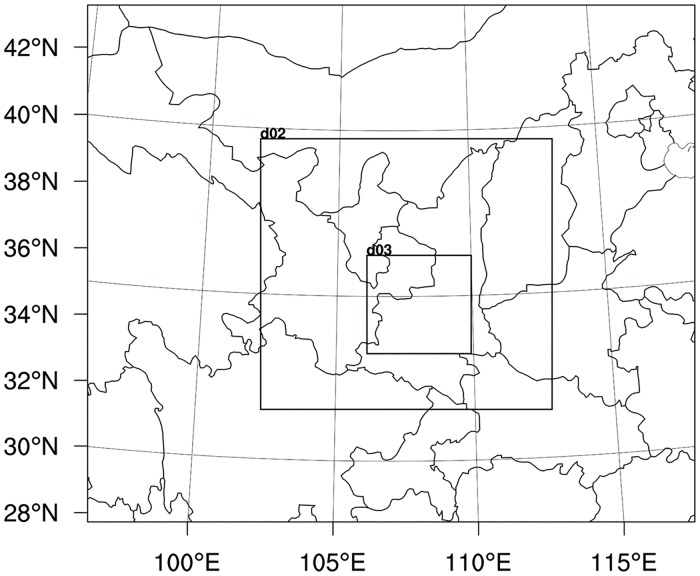
Nested computational domains in the WRF model configuration.

WRF is widely used for atmosphere simulation, due to its advanced dynamical model framework and reliable physical parameterization schemes. In this study, we adopt WRF version 3.4. A three-way nesting is employed (Fig 2), with a spatial resolution of 10.00, 3.33, 1.11 km and horizontal grid dimensions of 201×171 (zonal × meridional), 289×268, 310×292 respectively from domain 1 to 3. The innermost layer covers the Guanzhong Plateau. There are 27 full eta vertical layers. Integration step is set to 60s. The initial and boundary conditions are the FNL (Operational Global Final Analysis datasets) data provided by NCEP (National Centers for Environmental Predictions), with a resolution of 1°×1°spatially and 30 minutes temporally. As for physical schemes, YSU (Yonsei University) boundary layer scheme [38], WSM (WRF Single-Moment) microphysics scheme [39], RRTM (Rapid Radiative Transfer Model) [40] are adopted. For the land surface physical processes we concern, Noah land surface model [41] is adopted. In Noah LSM, potential evapotranspiration and latent heat are calculated by Penman-Monteith method. There are four layers on which soil temperature and water content are calculated, based on Thermal Diffusion Equation and Richards Equation respectively.

WRF Simulated single-point wind profile, temperature gradient, moisture gradient, and short wave radiation, soil moisture, soil heat flux, rainfall and other outputs are used to generate initial and boundary conditions for PKULM (described in the following section). PKULM ingests WRF output to update land surface state parameters, drive model operation, and output re-calculated surface flux and soil temperature simulation for each integration step.

### PKULM description and implementation

As previously discussed, the function of LSM is to simulate land˗atmosphere energy partition and substance exchange, which is essential to climate and ecosystem modeling. PKULM [32] went through lots of revision from former versions through years of development and it has extended biophysical and hydrological processes. Land Surface Exchange Model (LSEM) concludes vegetation shading and evapotranspiration [42].Modified Soil-Plant-Atmosphere Model (MSPAM) couples LSM to boundary layer scheme to study sensitivity of land surface and boundary layer processes to underlying surface characteristics. With the support of more flux observation in semi-arid area, soil heat transfer and plant biophysical processes have been improved [43, 44]. PKULM includes five basic modules: radiation transfer, turbulence, photosynthesis, soil heat diffusion and soil water transfer (no snow or frozen soil). It represents vegetation diversity based on 16 different Plant Functional Types (PFTs) with distinction of C3 and C4 plants.

### Governing equations in PKULM

Governing equations resolve water balance, energy balance, and heat transfer processes. For canopy water balance [45]:

$$\frac{dw_{v}}{dt}=q_{intr}-q_{drip}-E_{v}^{w}$$ (2)

Where *w*_*v*_ is canopy water content (mm), *q*_*intr*_ is water intercepted by canopy (mm s^-1^), *q*_*drip*_ is canopy drip (mm s^-1^), $E_{v}^{w}$ is evaporation from wetted fraction of canopy (kg m^-2^ s^-1^). 1 mm s^-1^ precipitation is equivalent to 1 kg m^-2^ s^-1^.

Ground water balance:

$$0=q_{rain}-q_{intr}+q_{drip}-q_{over}-q_{infl}$$ (3)

Where *q*_*rain*_ is rain rate (kg m^-2^ s^-1^), *q*_*over*_ is surface runoff (kg m^-2^ s^-1^), *q*_*infl*_ is infiltration (kg m^-2^ s^-1^).

Soil water content (Richard Equation) [46]:

$$\frac{\partial w}{\partial t}=-\frac{\partial q}{\partial z}-\frac{q_{root}}{\rho_{liq}}=\frac{\partial }{\partial z}\left(k_{h}\frac{\partial \psi }{\partial z}\right)+\frac{\partial k_{h}}{\partial z}-\frac{q_{root}}{\rho_{liq}}$$ (4)

Where *w* is soil volumetric water content (m^3^ m^-3^), *k*_*h*_ is hydraulic conductivity (m s^-1^), *ψ* is soil matrix potential (m), *q*_*root*_ is root water uptake rate (kg m^-2^ s^-1^), and *ρ*_*liq*_ is water density (kg m^-3^).

Canopy energy balance:

$$0=S_{v,net}+L_{v,net}\left(T_{v},\;T_{g}\right)-H_{v}\left(T_{v},\;T_{g}\right)-\lambda_{vap}E_{v}\left(T_{v},T_{g}\right)$$ (5)

Where *T*_*v*_ is canopy temperature (K), *S*_*v*,*net*_ is canopy absorbed solar radiation (Wm^-2^), *L*_*v*,*net*_ is canopy absorbed long-wave radiation (W m^-2^), *H*_*v*_ is sensible heat flux from canopy (Wm^-2^), *E*_*v*_ is transpiration from canopy (kg m^-2^ s^-1^), and *λ*_*vap*_ is heat of vaporization (J kg^-1^).

Ground energy balance:

$$0=S_{g,net}+L_{v,net}\left(T_{v},\;T_{g}\right)-H_{g}\left(T_{v},T_{g}\right)-\lambda_{vap}E_{g}\left(T_{v},T_{g}\right)-G\left(T_{g},T_{s}\right)$$ (6)

Where *T*_*g*_ is ground surface temperature (K), *S*_*g*,*net*_ is absorbed solar radiation by ground surface (Wm^-2^), *L*_*g*,*net*_ is ground absorbed long-wave radiation (W m^-2^), *H*_*g*_ is sensible heat flux from ground (Wm^-2^), *E*_*g*_ is evaporation from ground (kg m^-2^ s^-1^), and *G* is is soil surface heat flux (Wm^-2^).

Ground energy balance

Soil heat transfer:

$$C_{s}\frac{\partial T}{\partial t}=\frac{\partial }{\partial z}\left(k_{t}\frac{\partial T}{\partial z}\right)-C_{liq}\frac{\partial qT}{\partial z}$$ (7)

Where *T* is the soil temperature (K), *C*_*s*_ is the specific heat capacity of soil (J m^-3^ K^-1^), *C*_*liq*_ is the specific heat capacity of water (J m^-3^ K^-1^), and *K*_*t*_ is the soil thermal conductivity (J m^-1^ K^-1^ s^-1^).

### Description of selected physical process in PKULM

Considering the significant optical characteristics of canopy, solar radiation is divided into two wavebands: 0–700 nm and 700–2800 nm (spectra split conducted via SBDART transfer model [47]). Radiative transfer within vegetative canopy is calculated based on two-stream approximation [46]:

$$-\bar{\mu }\frac{dI↑}{d\left(L+S\right)}+\left[1-\left(1-\beta \right)\omega \right]I↑-\omega \beta I↓=\omega \bar{\mu }K\beta_{0}\text{exp}\left(-K\left(L+S\right)\right)$$ (8)

$$\bar{\mu }\frac{dI↓}{d\left(L+S\right)}+\left[1-\left(1-\beta \right)\omega \right]I↓-\omega \beta I↑=\omega \bar{\mu }K\left(1-\beta_{0}\right)\text{exp}\left(-K\left(L+S\right)\right)$$ (9)

Where *I***↑** and *I***↓** are the upward and downward diffuse radiative fluxes, *K* is the optical depth of direct beam per unit leaf and stem area, *μ* is the cosine of the zenith angle of the incident beam, $\bar{\mu }$ is the average inverse diffuse optical depth per unit leaf and stem area, *ω* is leaf scattering coefficient, *β* and *β*_0_ are up scatter parameters for diffuse and direct beam radiation respectively, *L* is the exposed leaf area index, and *S* is the exposed stem area index. Given the direct beam albedo $\alpha_{g,\Lambda }^{\mu }$ and diffuse albedo *α*_*g*,Λ_ of the ground on Λ waveband, these equations are solved to calculate the fluxes and per unit incident flux that is absorbed by the vegetation, reflected by the vegetation, and transmitted through the vegetation for direct and diffuse radiation for visible and near-infrared wave bands.

In vegetation, we assume the absorptivity and the emissivity to be equal. According to Stefan-Boltzmann Law and the definition of emissivity, the downward long-wave radiation below vegetation is

$$L_{v}↓=\left(1-\epsilon_{v}\right)L_{atm}↓+\epsilon_{v}\sigma T_{v}^{4}$$ (10)

Where *L*_*atm*_**↓** is the downward long-wave radiation from atmosphere (W m^-2^), *ϵ*_*v*_ is vegetation emissivity, *T*_*v*_ is canopy temperature.

PKULM adopts resistance model to calculate turbulence transfer [48, 49]. In the case of a vegetated surface, the sensible heat *H* and water vapor flux *E* are partitioned into vegetation and ground fluxes that depend on vegetation temperature *T*_*v*_ and ground temperature *T*_*g*_ in addition to surface temperature *T*_*s*_ and specific humidity *q*_*s*_. Assume the air within canopy have negligible capacity to store heat so that the sensible heat flux *H* between the surface at height *z*_0h_ + *d* and the atmosphere at height *z*_*atm*_ must be balanced by the sum of the sensible heat from the vegetation *H*_*v*_ and the ground *H*_*g*_.

$$H=H_{v}+H_{g}$$ (11)

$$H=-\rho_{atm}C_{p}\frac{\theta_{atm}-T_{s}}{r_{ah}}$$ (12)

$$H_{v}=-\rho_{atm}C_{p}\left(L+S\right)\frac{T_{s}-T_{v}}{r_{ih}}$$ (13)

$$H_{g}=-\rho_{atm}C_{p}\frac{T_{s}-T_{g}}{r_{bh}}$$ (14)

Similarly, assume the air within canopy have negligible capacity to store water vapor so that the water vapor flux *E* between the surface at height *z*_0*w*_ + *d* and the atmosphere at height z_atm_ must be balanced by the sum of the water vapor flux from the vegetation *E*_*v*_ and the ground *E*_*g*_.

$$E=E_{v}+E_{g}$$ (15)

Where

$$E=-\rho_{atm}\frac{q_{atm}-q_{s}}{r_{aw}}$$ (16)

$$E_{v}=-\rho_{atm}\frac{q_{s}-q_{sat}\left(T_{v}\right)}{r_{total}}$$ (17)

$$E_{g}=-\beta_{soil}\rho_{atm}\frac{q_{s}-q_{g}}{r_{aw}^{\text{'}}}$$ (18)

*r*_*aw*_ is the vapor resistance between canopy air and ambient atmosphere. $r_{aw}^{\prime }$ is the vapor resistance between ground to canopy air (sm^-1^). *r*_*total*_ is the total resistance to water vapor transfer from the canopy air and includes contribution from leaf boundary layer and stomatal resistance. Stomatal resistance (*r*_*s*_, s m^-1^) is calculated based on Ball-Berry half empirical model [50]:

$$\frac{1}{r_{s}}=m\frac{A}{c_{s}}\frac{e_{s}}{e_{i}}P_{atm}+b$$ (19)

where *m* and *b* are empirical coefficients, *P*_*atm*_ is the atmosphere pressure (Pa), *c*_*s*_ and *e*_*s*_ are the partial pressure (Pa) of CO_2_ and vapor above the leaf respectively, and the *e*_*i*_ is the partial pressure of vapor inside the stomata. *A* (*μmol m*^−2^*s*^*−*1^) is the assimilation rate of CO_2_, which is a function of canopy temperature, soil water potential, photosynthetically active radiation and partial pressure of CO_2_ and vapor. The calculation of stomatal resistance is vital to the simulation skill of canopy physiology and NPP.

According to resistance model, there is a relationship among *A*, *e*_*i*_,*c*_*i*_,*e*_*a*_ and *c*_*a*_:

$$A=\;\frac{c_{a}-\;c_{i}}{\left(1.37r_{b}+1.65r_{s}\right)\;P_{atm}}=\frac{c_{a}-\;c_{s}}{1.37\;r_{b}\;P_{atm}}=\frac{c_{s}-\;c_{i}}{1.65\;r_{s}\;P_{atm}}$$ (20)

To solve *r*_*s*_, define a function *h*(*c*_*i*_) = *c*_*a*_ − (1.37*r*_*b*_ + 1.65*r*_*s*_)*P*_*atm*_*A*. Thus *h*(*c*_*i*_) is the function of *c*_*i*_ and the eq (20) can be solved as a problem of curve crossing given point:

$$c_{i}=h\left(c_{i}\right)$$ (21)

in which we propose a bisection method to find a $c_{i}^{*}$ that fits eq (21) to ensure the robustness of the solution.

For one-dimensional vertical water flow in soils, the Richard’s equation [47] can be yielded by the conservation law of mass and Darcy’s law:

$$\rho_{liq}\frac{dw}{d\psi }\frac{\partial \psi }{\partial t}=\frac{\partial }{\partial z}\left[k_{h}\left(\frac{d\psi }{dz}+1\right)\right]-\frac{\partial }{\partial z}q_{root}$$ (22)

where *w* is the volumetric soil water content (m^3^ m^-3^), *q*_*root*_ is a soil moisture sink term (kg m^-1^ s^-1^), *k*_*h*_ is the hydraulic conductivity (kg m^-2^ s^-1^), and *ψ* is the soil matric potential (m).

In addition, several improvements in the calculation method have been embedded into PKULM. For example, we employ a new and effective bisection method to solve coupled photosynthesis and stomatal resistance model to ensure convergence, instead of the iteration method used in Community Land Model (CLM) that does not converge in low ambient vapor pressure [51].This method has been verified utilizing measured data at semi-arid sites where the model can cast reliable simulation; moreover, model comparison indicates PKULM surpasses Noah LSM modeling skill on heat flux, radiation, and soil characteristics [32].

### Methodology of starting PKULM

The initial conditions needed by PKULM include air temperature, specific humidity, wind speed at three directions, downward radiation both in long wave and short wave, ground heat flux, soil temperature and soil water content in different layers, liquid precipitation rate, atmosphere pressure, and CO_2_ concentration. The PFTs and their corresponding radiative properties and vegetation structure, turbulent properties, soil texture information have been prescribed. We provide its initial conditions by downscaling the WRF output and conduct land surface simulation cases with a temporal resolution of 30 minutes. A 10 hours’ spin-up period is already considered. Hereby due to scarcity of flux observation, we present qualitative comparison of the modeling skill between PKULM and Noah LSM. Due to many differences between PKULM and Noah, the simulation of two models may depart in value.

Sensible and latent heat flux are the fundamental parameters in quantifying terrestrial interactions. In case of vegetation existence, the total sensible heat fluxes (SH) between the atmosphere and the underlying surface consists of sensible heat flux from ground surface to canopy (SHG) and sensible heat flux from canopy to air (SHV). Likewise, the latent heat (LH) consists of latent heat ground (LHG) and latent heat canopy (LHV). Wind is vital to turbulence formulation, evaporation, and crop moisture transpiration. Furthermore, turbulence and water vapor are the key factors of SH and LH. Due to strong mixing effect by heat-induced turbulence, energy and moisture exchange are intensive and evaporation is strong at the interface of land surface and air.

## Results and Discussion

Judgment of DHW episode requires three indispensable meteorological elements, namely temperature, humidity, and wind speed. Hereby, to reveal the meso-scale circulation when DHW occurs, we present visualization of WRF simulated near-surface meteorological field over the district of interest in 2011 and 2013. We use simple downscaling method to process WRF output into the initial field driving PKULM at singe-column. Heat flux, radiation flux, and soil characteristics are simulated via PKULM to reveal the heat flux characteristics under the circumstance along with intensive evapotranspiration during the DHW episodes.

### Meteorological characteristics of two typical DHW episodes

**Fig 3:**
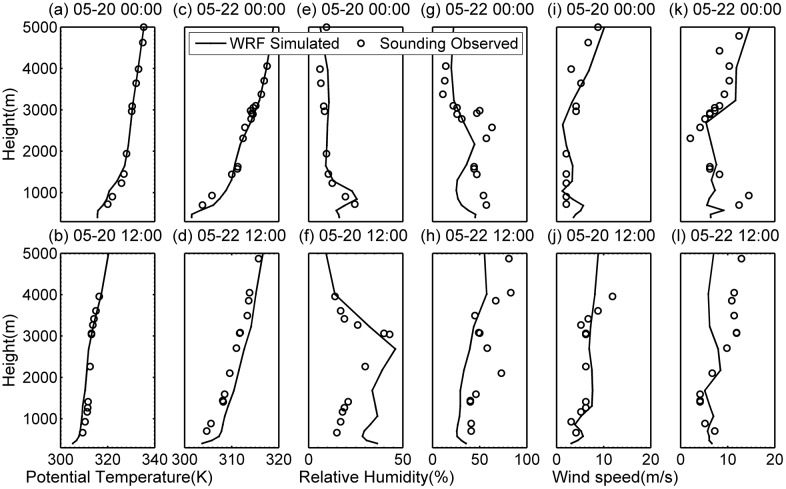
Comparison of observed sounding profile and in-situ output from WRF simulation respectively at 0000 and 1200 UTC on May 20, 22. (a-d) potential temperature, (e-h) relative humidity, and (i-l) wind speed.

WRF output during the first 10 hours has been excluded as spin-up. To verify the WRF simulation, we compare it with the sounding profile at Jinghe station (34°25'N, 108°58'E, which is the sounding base nearest to Wugong (34°16' N, 108°13' E). Verification helps us to determine simultaneous WRF output, including three critical factors potential temperature, relative humidity, and wind speed. The sounding data logs twice everyday, respectively at 0000 and 1200 UTC i.e. approximately 0800 and 2000 LST. The comparison is shown in Fig 3. WRF simulated potential temperature magnitudes and vertical profile are both in reasonably good agreement with sounding profile. Simulation shows that temperature lapse of daytime convective boundary layer and nocturnal stable boundary layer is clear. There are complicated factors affecting relative humidity, including but not limited to inhomogeneity of underlying surface and local moisture transfer. The simulation on relative humidity profile barely captures sounding observation features, especially at night, but the daytime humidity we concern here is comparatively reliable. As for wind speed, the simulation profile is generally close to sounding and the value stays in an acceptable interval given or taken from sounding.

**Fig 4:**
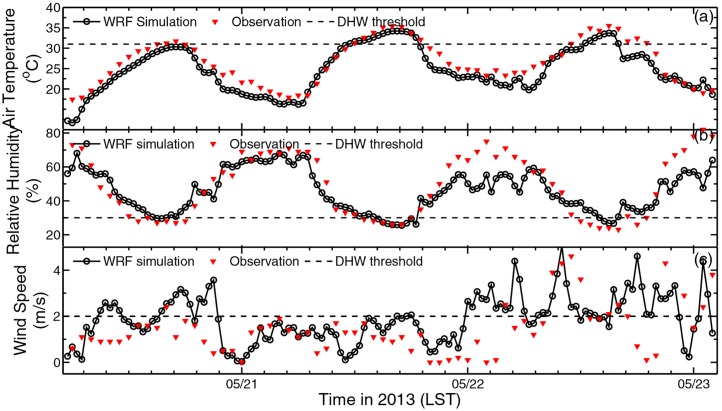
Comparison of WRF simulation versus observation at Wugong. (a) Temperature at 2m height, (b) relative humidity at 2m height and (c) near surface horizontal wind speed. In each panel figures, a horizontal dash line indicates the DHW threshold for each factors.

In view of DHW episode at station scale, a verification of the WRF simulation versus the in-situ observation on the three key factors must be considered. In Fig 4 we compares the diurnal variations in the observations at Wugong with those predicted by WRF for air temperature, relative humidity, and wind speed from May 19 to 23, 2013. Model values are extracted from the grid point nearest to Wugong. The temperature series reproduce a typical diurnal cycle with an increasing trend, and the values are in good concordance with observation with a mean relative error of ˗6.25%. The temperature simulation hits the DHW threshold (32°C) three times at 1400 LST in synchrony with observation. The WRF simulated relative humidity reproduces a diurnal cycle in strong concordance with temperature, but in an opposite trend, which is in agreement with common knowledge. The lowest humidity occurs at noon, with the value hit the DHW threshold three times in synchrony with observation. Simulation on relative humidity bias from observation with a mean relative error of ˗2.83%. Near surface wind is always variable and thus difficult to be reproduced precisely. This simulation of wind speed generally captures the increasing trend of this windy weather. Simulation on wind speed fluctuates in normal range consistent with observation. When actual wind did not hit DHW threshold (3ms^-1^) at 1400 on May 21, so does the simulation. Based on the above comparisons between simulations and observations from independent sources, we thus conclude that this simulation is reliable.

DHW actually is an areal phenomenon. Consequently background meteorological field and regional circulation during DHW episode need particular attention. The two disastrous DHW in 2011 (June 6 to 8) and 2013 (May 20 to 22) have representative prevailing wind field which is conductive to the formation of DHW. For both cases, the impact of topography on the spatial distribution of temperature and humidity is quite distinct.

**Fig 5:**
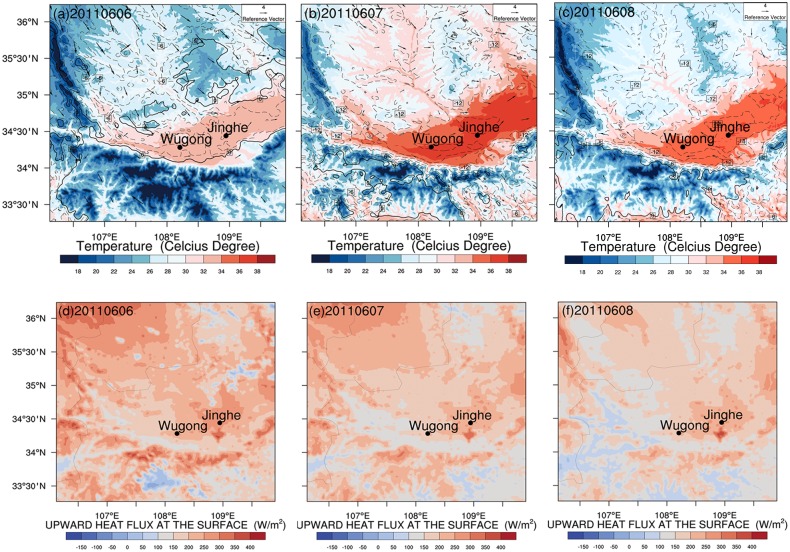
The near-surface meteorological features during 2011 DHW. (a-c) WRF Simulated wind, relative humidity, and temperature field near surface in domain 3 at 14:00 (LST) through June 6 to 8, 2011. Colored contour indicates temperature field at 2m height. Arrows indicate relative wind speed and direction at 10 m height. The black contour shows the field of difference between simulated relative humidity and 30% (i.e. the humidity criteria of dry-hot wind occurrence, solid line), and dashed line shows the negative value only, omitting positive value. (d-f) Color shading indicates WRF simulated near-surface sensible heat flux at corresponding time.

Fig 5 illustrates DHW Type A mechanism: prevailing northwest wind which is hot and dry brings large amounts of sensible heat to form DHW. Meanwhile, as time goes on (from Fig 5a–5c), northwest dry wind arrives at the plain, and ground surface absorbs solar radiation then emits intense long wave radiation to heat the upper air strongly. The accumulated heat makes this DHW episode sustainable to entrench over the interior plain and peaks on June 7 with widely spread high temperature and low humidity, worsen by the barrier effect of Qinling Mountain in downwind direction. Local heat from underlying surface is vital to raise air temperature. Ground sensible flux distribution (Fig 5d–5f) is another evidence of this mechanism. Noted that a contemporary high sensitive area below Jinghe spot is an bustling urban district. DHW fades gradually on June 8 along with the attenuation of background wind, while DHW still occurs. With continuously long-wave radiative heating, hot and dry air entrenches over the cropland, intensification of evapotranspiration leads to quick loss of crop’s carbohydrates even without strong wind.

**Fig 6:**
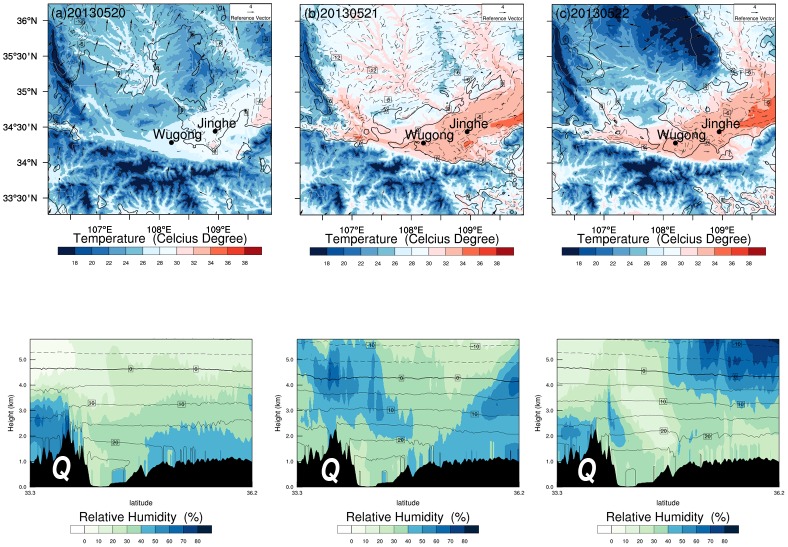
The near-surface meteorological features during 2013 DHW. (a-c) same as Fig 5, but through May 20 to 21, 2013. (d-f) Humidity section along 108.22°E meridional line. Q marks the Qinling Moutain.

DHW Type B is a typical dry adiabatic process. DHW is generated by the Foehn effect when southerly stream crosses the high mountain. It is illustrated in Fig 6a–6c), with the mountain-crossing stream marching, near-surface weather condition over the plain changes from cool and humid on May 20 to hot and dry later. Wind is weak over the plain because of mountain’s dynamic obstruction. The Foehn evolves from mountain-crossing stream and results in a favorable condition for DHW occurrence downwind. A humidity section at 1400 LST is shown in Fig 6d–6f) along a meridional line which crosses Wugong. It indicates an obvious dry adiabatic process when comparatively strong southerly stream loses its moisture in downwind after crossing the mountain and as it goes down to plain, DHW happens.

### Plot-scale simulations using PKULM

Following an overview of mesoscale characteristics of DHW, we conduct a land process simulation via PKULM to investigate the detailed heat transfer and plants’ responses to intensive evapotranspiration.

**Fig 7:**
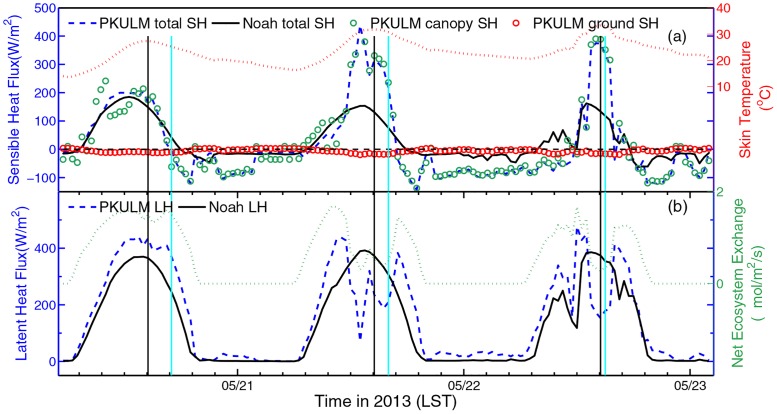
Heat flux simulation by WRF, PKULM respectively (left axis), with skin temperature and NEE (right axis) in 2013. Total sensible heat flux simulated by the two models, and (a) sensible flux of canopy and ground by PKULM, (b) latent heat flux simulated by the two models. For both two panels, the black meridional lines indicate the time when skin temperature peaks while the cyan lines indicate the time when in-situ observed 2 m temperature peaks (refer to previously in Fig 4a).

The diurnal cycles of SH (includes two ingredients) and LH are illustrated in Fig 7, with the diurnal cycle of skin temperature and net ecosystem exchange (NEE) for auxiliary analysis respectively. The skin temperature is the radiative temperature for upward long-wave radiation. The skin temperature modeled by PKULM casts a well diurnal variation with the increasing trend same as observed 2 m air temperature during DHW. After the total SH peaks, the skin temperature peaks later, indicating the ground heating effect to air.

PKULM simulated SH outnumbers a lot from Noah SH at noon on May 21 and May 22 (when the DHW happens) (Fig 7a). It does not fit the common sense from observation that the Noah total SH is decreasing as the DHW enhances. PKULM describes a more intense heat transfer than Noah. During DHW episode, it is reasonable that a high SH forming in ambient high temperature, resulting from fierce turbulence induced by large temperature gradient. Additionally, horizontal sensible heat transfer also possibly exits. Canopy bears a large proportion of total SH. Although SHV is high, SHG is negative occasionally in daytime, this is because of the canopy provides shaded cooling effect for the ground beneath it (vegetation coverage in PKULM is higher than Noah is according to FY-2 LAI product). Noticed that during the third cycle, there is a slight turning of skin temperature (a similar concave curve in observed air temperature) at 1000 LST, which indicates there may be some cloud alerting the incident shortwave radiation. Declining shortwave radiation also leads to change the “normal” SH produced by Noah at that time. Both two models’ simulations on upward radiation are declining at 1000 LST obviously (figure not shown).

Latent heat related to phase change of water is more than important in soil-plant-atmosphere interactive system. Moreover, the transpiration rate of plant is a key indicator of plant’s photosynthesis and carbohydrates accumulation [12]. In vegetated LSM case, it is common that simulated LH is approximately twice SH. Fig 7b depicts the constant diurnal variation of LH simulated by two models and the synchronous diurnal variation of NEE. The LH produced by Noah peaks smoothly every day, except in the third cycle when the cloud effects energy balance we have discussed previously. While PKULM LH produces a value-declining “valley” at everyday noon. This “valley” appears more evidently in the latter two-day cycles. It is known that plant stomata has a basic function to shut itself down to prevent unnecessary moisture from losing in hot and dry ambient, for example at sunny noon. When the stomata is closed, the leaf stops transpiration and absorbing ambient CO_2_, which leads to suspension of photosynthesis. Consequently, LH from canopy should decline correspondingly. This process has been legibly resolved in PKULM, the third-generation LSM with the support of Ball-Berry half empirical model for calculating stomatal resistance.

**Fig 8:**
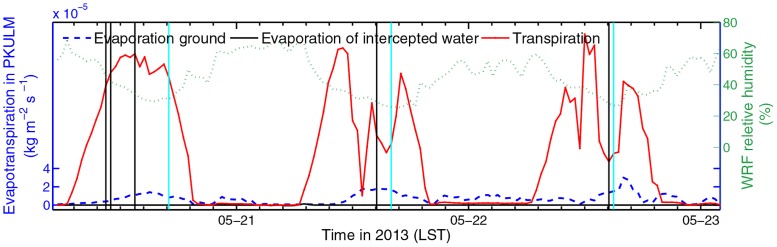
Evapotranspiration rate simulated by PKULM, including ground Evaporation, evaporation of water incepted by canopy, and transpiration of canopy in 2013 (left axis). In-situ WRF relative humidity (right axis). The black meridional lines indicate the time when skin temperature peaks while the cyan lines indicate the time when in-situ observed 2 m temperature peaks.

In PKULM, evapotranspiration consists of direct evaporation from ground, evaporation of water intercepted by canopy, and the plant transpiration. The average relative humidity from WRF output during 2013 DHW episode is 46.99%. Referring to simulated evapotranspiration illustrated in Fig 8, canopy transpiration constitutes the major proportion of evapotranspiration. In this semi-arid case in summer without precipitation, the water intercepted by canopy leaves is negligible. It is evident that on May 21 and May 22 1400 LST, the transpiration amount drops down quickly as a consequence of stomatal closure.

**Fig 9:**
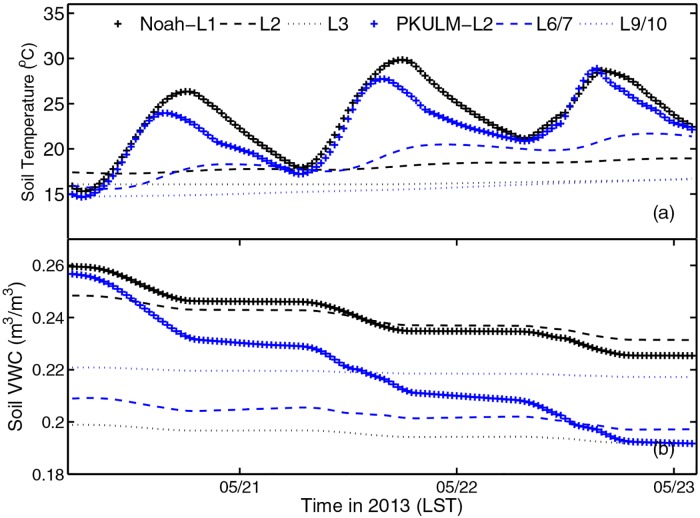
Soil temperature and volume water content in three soil layers (˗0.05, ˗0.25, and ˗0.7 m) simulated by PKULM and Noah and their comparison in 2013. (a)soil temperature, (d)soil volume water content (VWC).

Soil temperature and soil volume water content (VWC) at four soil layers simulated by PKULM and Noah are shown in Fig 9. There are 18 layers of soil configured in PKULM, with a depth span of ˗0.025, ˗0.05, ˗0.075, ˗0.1, ˗0.15, ˗0.2, ˗0.3, ˗0.4, ˗0.6, ˗0.8, ˗1.2, ˗1.6, ˗2.4, ˗3.2, ˗4.8, ˗6.4, ˗9.6, and ˗12.8 m respectively. The gird distribution is dense-to-sparse from near surface to deep down in soil. We interpolate PKULM soil temperature and VWC on three out of four layers at center depths of Noah soil layer (˗0.05, ˗0.25, ˗0.7, and ˗1.5 m) to compare. The temperature and moisture at ˗1.5m layer are negligible due to its nearly no variation. Both models reflect a diurnal variance yet in different value and amplitude at near-surface layers. Soil temperature is driven by air temperature but in a lagged phase with reference to air temperature (Fig 4a). At 0.3 m and 0.6 m depth, PKULM still reproduce the more realistic undulating diurnal cycle of soil temperature, while soil temperature from Noah shows barely any fluctuation. PKULM soil temperature simulation exceeds Noah simulation at all layers (Fig 9a), while its VWC is lower than Noah VWC at all layers (Fig 9b). Soil temperature bias can be explained as: during this DHW episode, although increasing air temperature forces high soil temperature, denser vegetation coverage (higher LAI) has been introduced into PKULM, which alters surface albedo and the denser canopy provides a shaded cooling effect. Conceptually, for upper layer soil, direct ground evaporation is the dominate factor for the soil water content, while for deeper layer soil, transpiration rate or the amount of water uptaken by plant root dominates. Stomatal closure induces transpiration declines, then canopy root uptakes less water from soil. Therefore, the soil water content will be protected from losing too quickly from root effect view. This conceptual mechanism is proved in Fig 9b. PKULM reproduces a faster declining near-surface soil VWC than Noah due to the intense ground transpiration. While at ˗0.25 m layer, PKULM casts a slower declining soil VWC due to the suppressed root uptaking effect on soil water.

**Fig 10:**
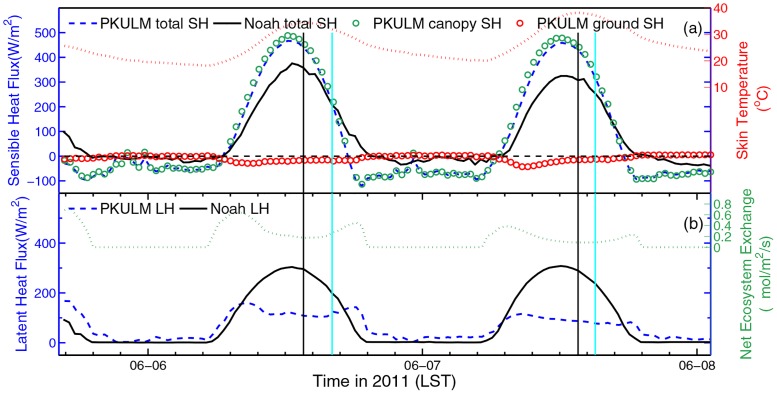
Similar to Fig 7, but in 2011.

**Fig 11:**
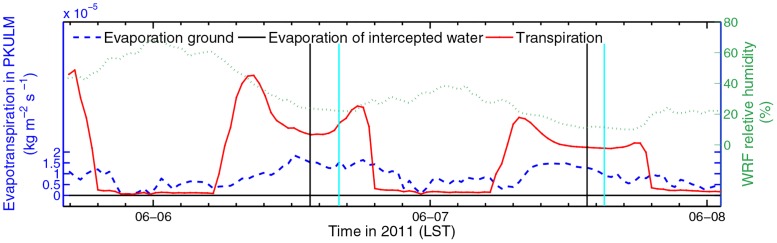
Similar to Fig 8, but in 2011.

We investigate the 2011 DHW episode in similar logic flow. What makes 2011 DHW episode different from the one in 2013 is that the DHW is already severe in June 6, 2013 (see Table 1). In addition, there is barely any SH advection because this DHW is caused by Foehn. As illustrated in Fig 10, the PKULM simulated SH outnumbers Noah simulated SH only in a range of 100 W m^-2^. The canopy also bears tremendous SH. The Noah simulated LH remains a constant diurnal cycle and peaks at 300 W m^-2^ yet. But referring to Fig 11, it’s obvious that in 2011 episode, the average air relative humidity is 30.79% with a rushed declining trend, which is much lower than that in 2013 (46.99%). The evapotranspiration could not be high with already losing lots of moisture both from soil and plant, although under hot and dry circumstances. In such a low ambient relative humidity and corresponding lower soil VWC (the soil VWC is only 0.2 m^3^ m^-3^ with barely any variance, figure omitted), the lower transpiration and LH simulated by PKULM is reasonable. And the stomatal closure process is evident, too.

## Conclusions

Dry hot wind (DHW) is a frequent hazard to Chinese mainland food production. In this paper, we investigate the formation mechanism on synoptic scale and from the view of terrestrial interactions concerning DHW. The novelty of this research consists of revealing two possible mechanisms of DHW formation and simulation on terrestrial interactions via a new land surface model PKULM. Based on reliable synoptic simulation by WRF, two typical mechanisms of DHW occurrence are revealed: one is that prevailing heated dry wind stream forces the formation of DHW along with intense sensible heating; the other one can be explained as a dry adiabatic process. For detailed terrestrial interaction modeling, we present a third-generation LSM PKULM with advanced framework, better solving methods, and augmented physiological descriptions. PKULM performance has been verified reliable with solid observation over semi-arid grassland and wheat land 100km away from Wugong.

How land–atmosphere system responds under the circumstances of intense evapotranspiration during DHW episode is revealed on basis of PKULM simulations. Leaves take considerable amounts of sensible heat flux. The stomatal closure at noon is legibly evident according to latent heat and transpiration simulations. NEE is in evident correlation with transpiration. The upper layer soil dries out quickly due to direct ground evaporation; while deeper soil loses moisture slowly due to suppressed transpiration rate and suppressed root uptaken effect resulted from stomatal closure. However, in extreme dry scenario, the transpiration cannot be high, and the soil cannot lose moisture any more. These findings provide researchers with an insight into the processes of heat transfer, soil hydrology, radiation, and physiology within land–atmosphere system. In addition, they are useful for further investigation of NEE and gross primary production (GPP) modeling, irrigation configuration and improving crop models.

Land–atmosphere system involves multiple complicated processes; there remain quite a few uncertainties. Development of novel LSMs is of great necessity for ensemble land surface hindcast and forecast. Simultaneously, increased complexity of new generation models requires innovative field observation and integrative metrics by which the model interacting features can be assessed. We look forward to acquisition of more eddy covariance observation to validate and modify the PKULM. With further validation based on long-term observation on various underlying surface, we have envision to employ PKULM as an optional LSM scheme into mesoscale models like WRF to help take an insight into issues concerning crop physiology in a mesoscale framework.

## Supporting Information

S1 Fig Spatial distribution of FY-2 derived Leaf Area Index over the district of interest in summer. (TIF)
