# Supplementary figures and images for: Numerical Study on the Stomatal Responses to Dry-Hot Wind Episodes and Its Effects on Land-Atmosphere Interactions
Source: PLoS One. 2016 Sep 20;11(9):e0162852. doi: 10.1371/journal.pone.0162852 (PMC5029913; doi:10.1371/journal.pone.0162852)

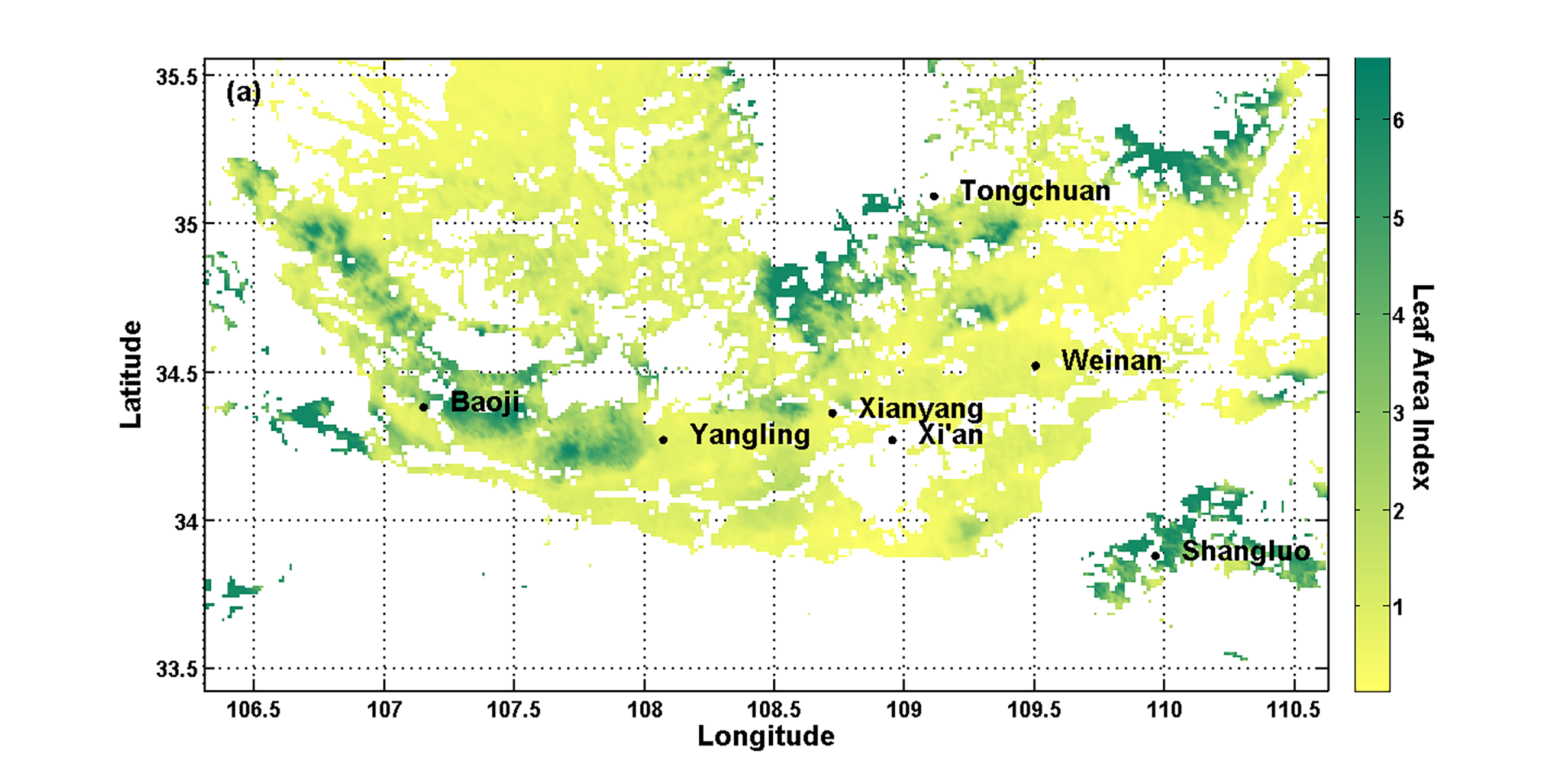

Supplement: S1 Fig — (TIF) [file pone.0162852.s001.tif]
